# Temporomandibular joint space variation and masticatory muscle activation during clenching with full versus partial covering occlusal splints

**DOI:** 10.1007/s00784-024-05980-0

**Published:** 2024-10-10

**Authors:** Annika Seiler, Nenad Lukic, Mutlu Özcan, Marina Kazimi, Moody Kaldas, Luigi M. Gallo, Vera Colombo

**Affiliations:** Clinic of Masticatory Disorders and Dental Biomaterials, Center for Dental Medicine, University of Zurich, Plattenstrasse 11, Zurich, CH-8032, Switzerland

**Keywords:** EMG, Motion capture, MRI, Occlusal splints, Temporomandibular disorders, Temporomandibular joint

## Abstract

**Objectives:**

Occlusal splints are the main therapeutic choice in the treatment of temporomandibular disorders (TMD). However, their precise working mechanism is unclear. This study aimed to compare the biomechanical effect of three commercially available splint designs (full covering splint, anterior bite splint and posterior bite splint) during biting in a sample of healthy subjects.

**Materials and methods:**

Magnetic resonance imaging (MRI) was combined with jaw tracking to measure the minimal intraarticular distance (MID) of 20 human temporomandibular joints (TMJ) whilst simultaneously recording the electromyogram (EMG) of the masticatory muscles. The changes caused by clenching with a bite force of 100 N without splint (baseline) and on each splint were calculated. Repeated measures ANOVA was performed on the means of the MID variations and EMG amplitudes.

**Results:**

Clenching on the anterior bite splint resulted in two times less activation of the anterior temporalis muscle than baseline (*p* = 0.003), full covering (*p* = 0.011) and posterior bite splint (*p* = 0.011). MID was reduced by clenching in all conditions, but the reduction was almost three times larger with the anterior bite splint compared to no splint (*p* = 0.011). The full covering splint and the posterior bite splint did not differ significantly in EMG activation of both masseter and temporalis muscles and MID variation.

**Conclusions:**

This study showed that splint designs have a different impact on the MID and EMG activation while clenching. The anterior bite splint had a greater impact on the reduction of the muscle activation, whereas clenching on the anterior bite splint led to bigger reduction of MID and thus had the greatest influence on alteration in the condylar position.

**Clinical relevance:**

The design of the splint can affect MID and muscle activation and is a variable to consider in the treatment of patients with TMD according to their symptoms.

## Introduction

Temporomandibular disorder (TMD) is a term used to describe a group of clinically relevant conditions affecting the masticatory muscles, the temporomandibular joint (TMJ), and their associated structures [1]. Symptoms such as decreased or altered mandibular range of motion, joint crepitus or clicking, headache or pain in the orofacial region can occur [2]. These symptoms often radiate into adjacent body regions such as the ears [3, 4], the head, the neck and the cervical spine [5]. Since the described symptoms have a great impact on a patient’s quality of life, TMD represents a significant public health problem.

A considerable variation in the prevalence of TMD pain has been reported in the literature, especially before the introduction of standard diagnostic guidelines such as the Research Diagnostic Criteria for TMD (RDC/TMD) in 1992 [6] and the Diagnostic Criteria for TMD (DC/TMD) in 2014 [7–10]. Recently, a systematic review by Valesan et al. showed that the percentage of TMD among adults diagnosed following the RDC/TMD or DC/TMD guidelines is estimated to be approximately 31% and 11% for children [11]. Symptomatic TMD patients are spead over a wide age range, but exhibit peak appearance at the age of 20 to 40 [8, 11, 12]. The etiology of TMD is complex and multifactorial. Physical, structural, physiological, postural, genetic and psychological factors can contribute to the disorder [13].

Due to incomplete knowledge in the aetiology and pathogenesis of TMD, the diagnosis and management pose a great challenge [1, 2, 13]. The main treatment options include splint therapy [14], psychotherapy [15], medication [16], physical therapy [17] and surgery [18]. Occlusal splint therapy is a common approach in the treatment plan of a dental practice, although its precise mechanism of action remains unclear [19, 20]. However, many studies agree on the important role of occlusal splints in the therapy of TMD [14, 21, 22]. Over the last decades, several splint designs have been conceived and employed for the management of TMD [19]. The most commonly used occlusal splint is the full covering splint, also known as Michigan splint or stabilization splint. It consists of an individually manufactured appliance, covering all teeth in the dental arch and designed so that each antagonistic tooth makes one contact with the splint. It can be produced for either the maxillary or the mandibular arch but is usually positioned on the maxillary arch [23]. Conversely to full covering, partially covering splints only cover a portion of the dental arch and they are categorized as anterior or posterior bite splints, depending on the covered region of the dental arch. Anterior bite splints are appliances that cover generally two to four incisors on the maxillary or mandibular arch. Commonly, these splints are made from a prefabricated matrix which can be adapted to an individual anterior bite splint. They are used for the treatment of bruxism, TMD, cervical pain and different types of headaches and they are commercialized under several brand names [24]. Posterior bite splints or pivot appliances are individualized splints that make occlusal contact only with the most posterior teeth, leaving the anterior teeth out of occlusion. For this type of splints, apart from the individually manufactured designs, standard vacuum-formed appliances are also present in the market. In particular, the Aqualizer^®^ (Jumar Corporation, Bainbridge Island, WA, USA) advertised as a cost- and time effective splint with water-filled elastic pads which showed good clinical results in treatment of painful TMD [25, 26].

Hypothesized explanation models for understanding the mechanism of action of the occlusal splint in TMD include the change in condylar position, leading to a reduction or change of joint loading. Another mechanical explanation can be the change in occlusal contacts that improve the distribution of forces, an increase of vertical dimension leading to more stretching of the masticatory muscles. Occlusal change leading to an altered and reprogrammed peripheral feedback from the periodontal ligament is the so-called neurological explanation. In few previous studies, it was demonstrated that the insertion of a stabilization splint led to a change in condylar position [27, 28] or a significant decrease of intra-articular pressure in the upper TMJ compartment [29] or altered electromyographic (EMG) activity [30]. However, the biomechanical effects of occlusal splints for the temporomandibular joint under parafunctional stress remain poorly understood. Therefore, it is difficult to achieve a targeted treatment for TMD [1].

To further explore the effects of occlusal splint in therapy, this study analyzed the impact on TMJ biomechanics of three different occlusal splint types (the full covering - or stabilizing - splint, the anterior bite splint and the posterior bite splint) using electromyography and dynamic stereometry where the latter method can relate mandibular movements to TMJ anatomical structures by combining three-dimensional reconstructions of the TMJ anatomy with jaw motion recordings [31, 32]. Furthermore, by measuring the condyle-fossa distance, the minimal intraarticular distance (MID) can be determined, which reflects the TMJ loading state [28, 33].

The aim of this study was to measure the effect of subsequent clenching with 100 N on three different occlusal splints on the TMJ intra-articular space variation and on the EMG of the masticatory muscles. The null hypothesis was that there would be no difference in masticatory muscle activation and joint space during clenching by using the three different occlusal splints.

## Materials and methods

### Subjects

Twelve study participants were recruited among students of the Center for Dental Medicine and University Hospital Zurich. Inclusion criteria were: age between 18 and 99 years and willingness to participate in the study. Excluded from the study were subjects with signs or symptoms of TMD according to DC/TMD diagnostic criteria, an open bite, a negative overjet or overbite more than 3 mm, systemic diseases, contraindications for magnetic resonance imaging (MRI) (implanted metal or medical devices, claustrophobia, big tattoos) or a current or planned pregnancy during the study. Subjects who could not follow procedures, lacked sufficient comprehension of the German language or were unable to give consent were also excluded. To be enrolled in the study, an informed consent was signed by all participants prior to the beginning of the experimental session. The Ethics Committee of the state of Zurich approved the study protocol (KEK-ZH- Nr. 2019–02159).

### Splint design

**Fig. 1:**
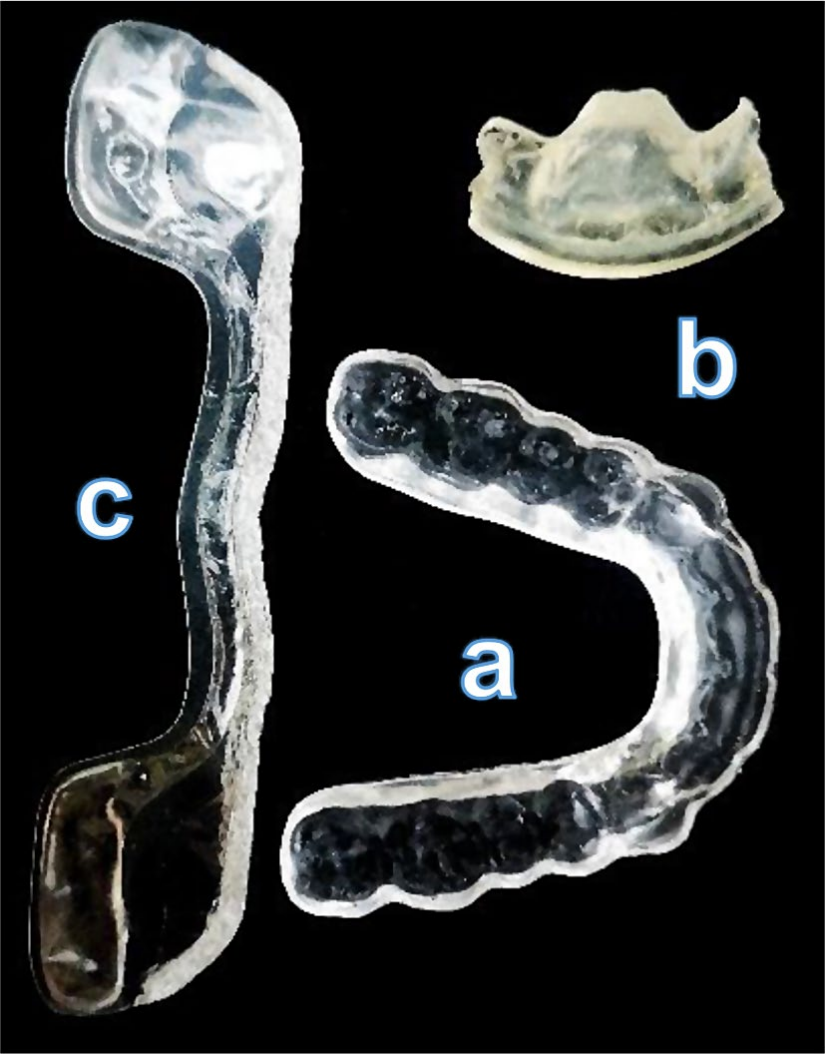
Top view of the three splints which were used. (**a**) Full covering splint (**b**) Anterior bite splint (**c**) Flattened-out Posterior bite splint

Three different splint designs were used in the study: full covering splint, anterior bite splint, posterior bite splint (Fig. 1). The full covering and the anterior bite splint were manufactured individually for each participant, whereas for the posterior bite splint, a standard solution was selected. All selected splints were commercially available solutions. The full covering splint and the matrix for the anterior bite splint were produced by the same manufacturer (Orthotixx Dental AG, Reichenburg, Switzerland) and were designed with the same thickness of 1.5 mm. The full covering splint was manufactured from PMMA blanks (DDBioSplintPHI, Dental Direkt GmbH, 32139 Spenge, Germany). The anterior bite splint, commercialized under the name FOS, consisted of a prefabricated matrix, which can be adapted to an individual splint. The prefabricated matrix was made of polyester copolymer, and the individual fitting to the subject was executed by using an auto-polymerized resin material (Unifast III Monomer, GC Asia Dental Pte Ltd, Singapore) mixed with its transparent powder  [24]. Besides the individually manufactured splint types, a commercially available standard posterior bite splint was included in the comparison. The splint is commercialized under the name Aqualizer^®^ (Jumar Corporation, Bainbridge Island, WA, USA) and is advertised as a cost- and time effective splint with water-filled elastic pads which is favorably indicated for painful TMD [25, 26]. It is available in different sizes and thicknesses (1 mm / 2 mm / 3 mm). In this study, we used the average size (Aqualizer^®^ Ultra) with a thickness of 2 mm.

### Study procedure

**Fig. 2:**
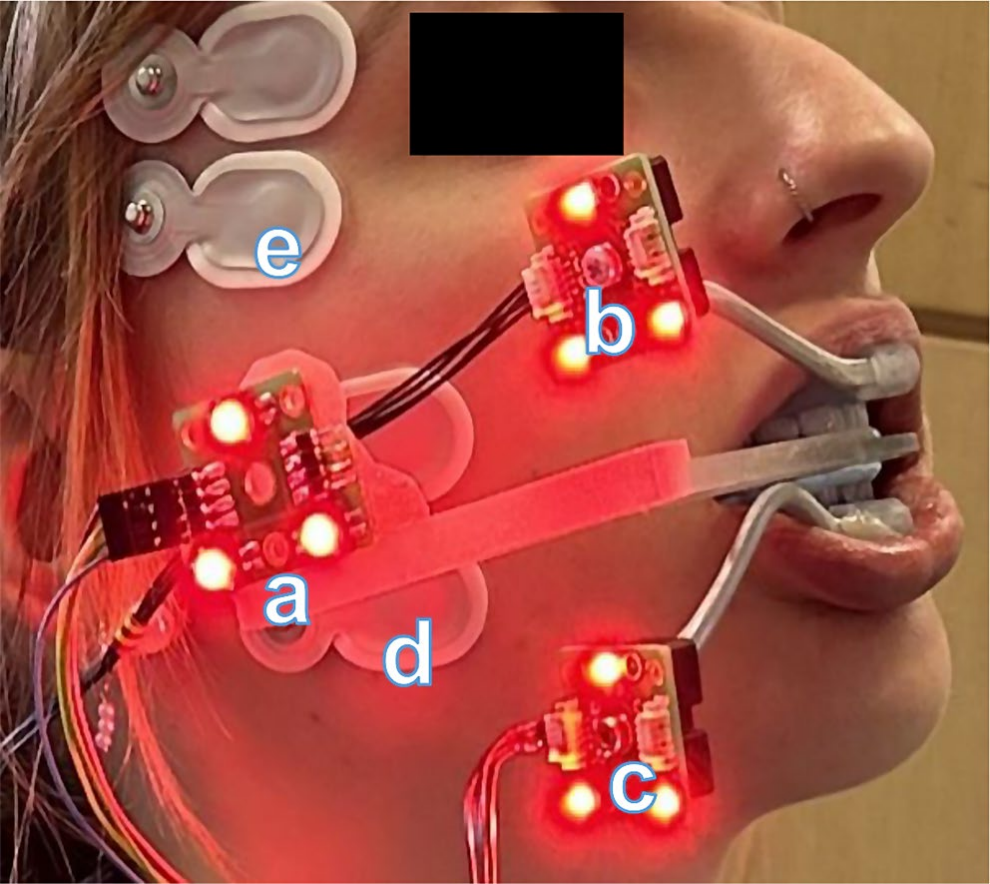
Jaw tracking with (**a**) facebow holding to the reference target frame and the anatomical references for MRI in a fixed geometrical relation. (**b**) Maxillary target frame with the personalized service-splint attached to the teeth. (**c**) Mandibular target frame with the personalized service-splint attached to the teeth. Surface electrodes (**d**) on the masseter and (**e**) the anterior temporalis muscle

Study participants were invited to attend three appointments in which their anatomical, EMG, and kinematic data were collected. The first appointment took place at the MR Center of the University Hospital Zurich, where an MRI of the participant’s TMJs was taken. During the MR session the participants were biting on a monobloc carrying an individual facebow linked to three spheres, which were used as geometrical references in the protocol. (Fig. 2). The monobloc was covered with Blu-Mousse^®^ (VBS Bite Registration Material, Parkell Inc., USA) to allow for a precise repositioning of the jaw in the following steps of the protocol.

A parasagittal MRI scan of the TMJs was obtained for each participant (Ingenia 1.5 T scanner with a dStrem Flex S coil, Koninklijke Philips Electronics N.V., the Netherlands). Any findings or signs of TMD in the MRI results led to the exclusion from the study. The articular regions of fossa and condyle were contoured in the 2D MR images, and 3D digital models of their surfaces were obtained using a medical image processing software (Amira™ v. 6.5, TFS, Waltham MA, US).

The second appointment took place at the Center for Dental Medicine of the University of Zurich, where the participants were examined for diseases and pathologies of the masticatory muscles and the temporomandibular joint by means of the DC/TMD protocol [10]. If the inclusion criteria were met, a digital impression of the maxillary and mandibular dental arches of the participant was obtained using an intraoral scanner (TRIOS, 3Shape, Denmark). The digital impressions of the teeth were sent to an external dental technician to design the full covering splint. Furthermore, in preparation for the experimental session, two personalized service-splints were designed in house with a computer assisted design (CAD) software (Rhino 6^®^; McNeel Inc., Seattle WA, USA; https://www.rhino3d.com) and 3D-printed (Objet Eden 260 V™; Stratasys, Eden Prairie 162 MN, USA). These custom-made service-splints were used as holder for the target objects (triangular target frames, TTF) acquired by the cameras during the motion tracking. They are designed to cover only the buccal surface of the upper and lower front teeth (canine to canine) and not to interfere with the occlusal splint position during the motion-tracking session (Fig. 2).

The experimental session with clenching movements took place on the day of the third appointment. To collect the motion data, a custom-built optoelectronic motion-tracking device, developed at the Center of Dental Medicine of the University of Zurich was used. The device is composed of three infra-red cameras recording the position in space of triplets of light emitting diodes (LEDs) with a frequency of 200 Hz and a spatial resolution of 5 μm. Each triplet, called target frame, represents a moving object in space. To measure the position of the mandible and the head, each of the two 3D-printed personalized service-splints carrying a metal rod with a triangular target-frame was attached to the maxillary and mandibular front teeth with photo-polymerized compomer (ULTRA BAND-LOK, Reliance orthodontic products, USA). This allowed to calculate the motion of the mandible relative to the fossa. Furthermore, at the beginning of the experimental session, a reference recording was carried out with the participant gently biting on the reference facebow used during the MR session (Fig. 2), equipped with an additional target-frame. This third object served as a cross-reference between the anatomical and kinematic session coordinate systems and allowed for the subsequent animation of the anatomical features of the TMJ [31].

To record the masticatory muscle activity, self-adhesive, pre-gelled Ag/AgCl electrodes (Type Neuroline 720 00-S/25, Ambu, Ballerup, Denmark) were attached bilaterally to the masseter and anterior temporalis muscle, while one reference electrode was placed on the left mastoid bone (Fig. 2). Before electrode application, the skin was prepared with a peeling cream (Lubex peeling^®^, Permamed AG, Therwil, Switzerland) and electrode solution (signa^®^ spray, PARKER LABORATOIRIES INC., New Jersey USA) to reduce skin impedance. For correct electrode placement, the muscles were palpated, and the electrodes were placed along the main fiber direction of the muscles. Male subjects were advised to shave themselves before attending the appointment to enhance the adhesion of the electrodes.

### Experimental procedure

At first, participants were instructed how to perform the required movement tasks, then the manufactured full covering splint was tested and, if necessary, adapted chairside to the subjects’ anatomy by removing the splint material with an acrylic bur in case of pressure points. Afterwards, the anterior bite splint was fabricated chairside and participants were asked to perform different oral tasks.

At first, subjects had to clench on a custom-built bite-force sensor positioned between the left first molars with 100 N. Thus, the muscle activation was defined and used as a reference for the following clenching task. Four conditions were examined in a randomized fashion: no splint, full covering splint, anterior bite splint, posterior bite splint. Subject were requested to keep their teeth in contact (maximum intercuspation, ICP) for two seconds without applying any force, and then clench starting from this position with 100 N contraction effort for approximately three seconds. The task was repeated three times for each observed condition.

### Data analysis

MR images were used to acquire the individual shape of the mandibular condyle and the fossa of the TMJ. To provide a dynamic, non-invasive in vivo view of the TMJ, it was necessary to link the anatomical structures from the MRI to the Optoelectronic Tracking System (OPTIS) using a proprietary software developed at the Center for Dental Medicine. The obtained data were used to record the movements of the lower jaw and the processes in the TMJ. By means of the 3D digital model of the TMJ it was possible to identify the minimum intra-articular distance (MID) as the average of the 30 smallest condyle-fossa distances, measured between the polygonal vertices of the model meshes at each time step of mandibular motion, as described in previous studies (23, 26–28).

### Statistical analysis

IBM SPSS^®^ Statistics version 29 software (IBM Corporation, Armonk, NY) was used for statistical analysis. The mean values of MID and EMG amplitudes of the masseter and temporalis muscles were determined with the subject holding the teeth in maximum intercuspation (ICP) and during clenching with 100 N and the differences between ICP and clenching was calculated for both left and right joints. The mean of the three repetitions was calculated and the left-right values were compared. Normality tests (Shapiro-Wilk) showed that the variables were not normally distributed. Therefore, the comparison between left and right condyle was carried out with non-parametric Wilcoxon test for related samples. Since there were no statistical differences found between the sides, the mean of right-left data was calculated. Descriptive statistics was performed on the means of the variations; mean, standard deviation, median, maximum, minimum and range were calculated for all four observed conditions (no splint, full covering splint, anterior bite splint, posterior bite splint).

The variables ΔMID, ΔEMGTemp, ΔEMGMass (means of left/right of the variations between ICP and clenching) were used to assess the differences among the observed conditions. Normality tests were carried out on the variables. Non-parametric Friedman tests for related samples were performed on the sample. The significance level was set at α = 0.05.

## Results

From the initially recruited twelve healthy volunteers, six male and six female, two female subjects were excluded during the study due to signs of TMD and failure in the graphical segmentation of the joint. The final pool of participants consisted of six male and four female aged between 22 and 35 years (mean: 24 ± 4 years). Therefore, a total of 20 TMJs were analyzed in the study.

**Fig. 3:**
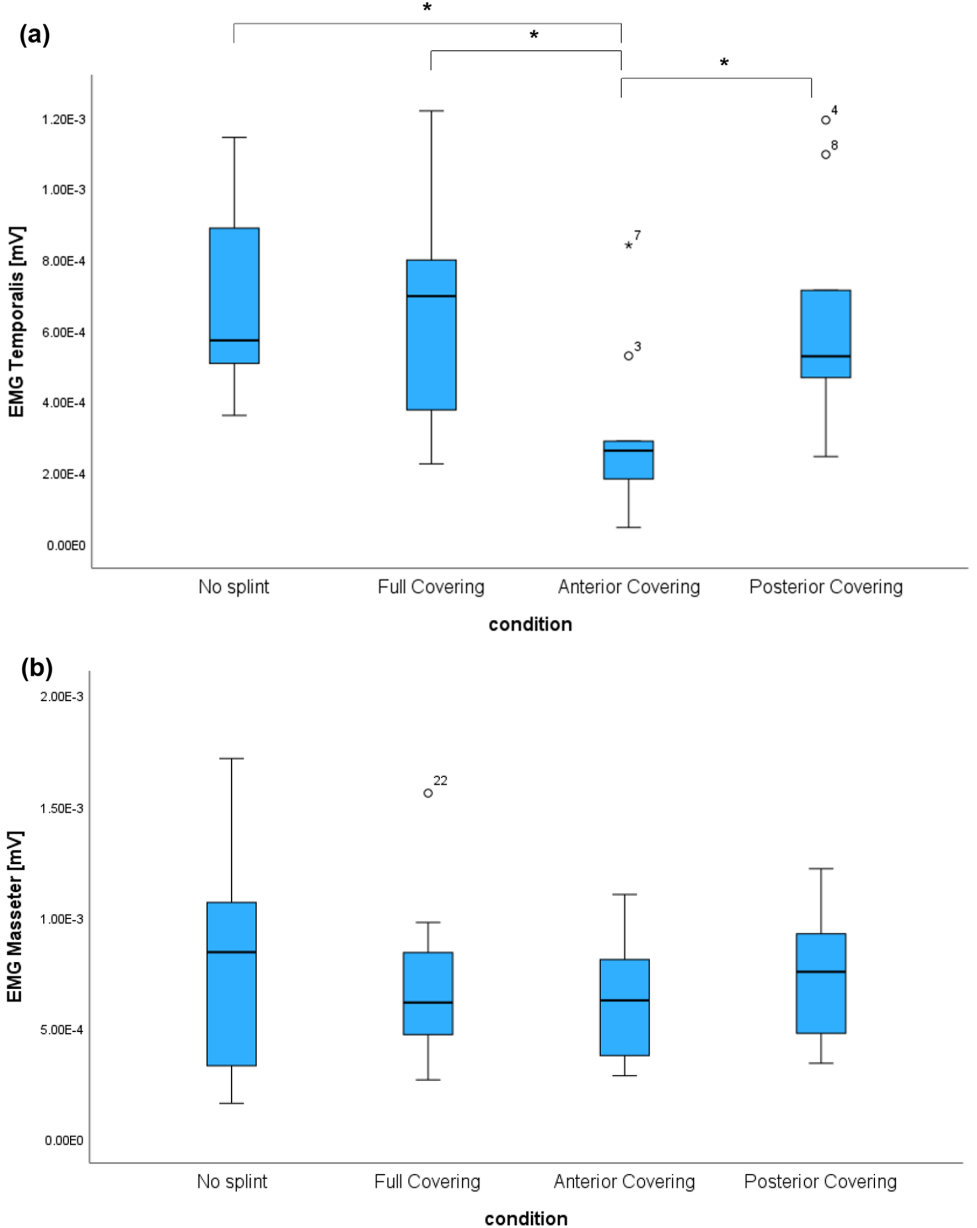
Graphic representations of the parameters describing ΔEMG (**a**) of the anterior temporalis muscle and (**b**) of the masseter muscle between ICP and clenching with bite force of 100 N while wearing no splint, a full covering splint, an anterior bite splint or a posterior bite splint

Clenching with bite force of 100 N without splint, full covering splint and posterior bite splint resulted in a twice as high activation of the temporalis muscle compared to the anterior bite splint (*p* = 0.003, *p* = 0.011, *p* = 0.011 respectively) (Fig. 3a, Table 1a).

**Table 1:** Mean ± standard deviation (SD), median and range with max-min of the average left and right of (a) ΔEMG Temporalis [µV], (b) ΔEMG Masseter [µV] and (c) ΔMID of the joint [mm] between ICP and clenching with bite force of 100 N

Condition	No splint	Full covering splint	Anterior bite splint	Posterior bite splint
**(a)**				
Mean ± SD	0.67 ± 0.08	0.63 ± 0.10	0.30 ± 0.07	0.63 ± 0.10
Median	0.57	0.70	0.26	0.53
Range [max - min]	0.78 [1.14–0.36]	0.99 [1.22–0.22]	0.80 [0.84 − 0.05]	0.95 [1.19–0.25]
**(b)**				
Mean ± SD	0.77 ± 0.16	0.68 ± 0.12	0.63 ± 0.09	0.74 ± 0.09
Median	0.84	0.61	0.62	0.75
Range [max - min]	1.56 [1.72 − 0.16]	1.29 [1.56 − 0.27]	0.82 [1.10–0.28]	0.88 [1.22–0.34]
**(c)**				
Mean ± SD	0.25 ± 0.071	0.35 ± 0.09	0.68 ± 0.07	0.42 ± 0.11
Median	0.20	0.39	0.56	0.32
Range [max - min]	0.80 [0.82 − 0.03]	0.98 [0.67 - -0.31]	0.55 [1.04–0.49]	1.20 [1.29 − 0.09]

The four different conditions led to a similar increase (*p* > 0.05) in the EMG activation of the masseter muscle, when changing from ICP to clenching with 100 N (Fig. 3b, Table 1b).

**Fig. 4:**
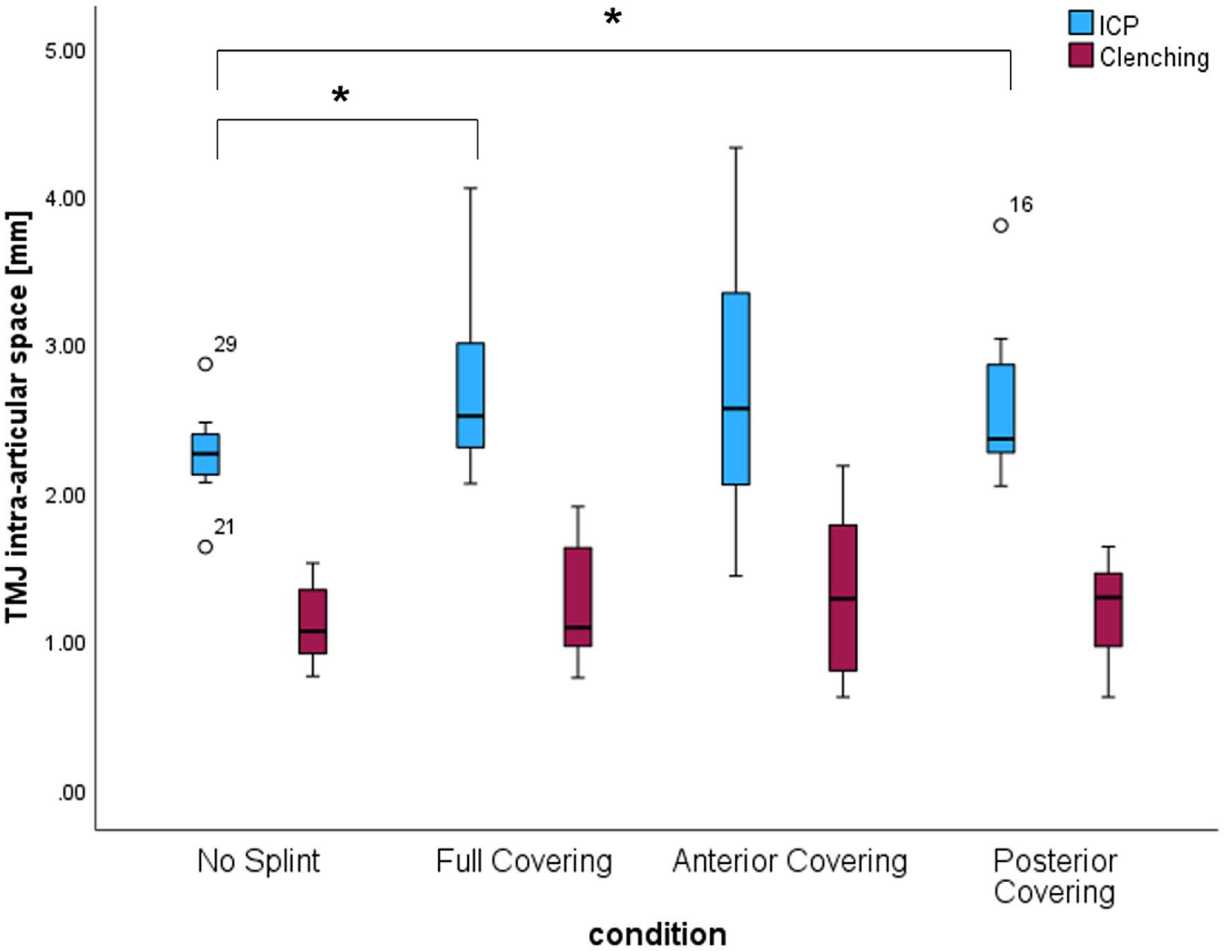
Graphic representation of the parameters describing the joint intra-articular space in ICP and during clenching with bite force of 100 N while wearing no splint, a full covering splint, an anterior bite splint, or a posterior bite splint

**Fig. 5:**
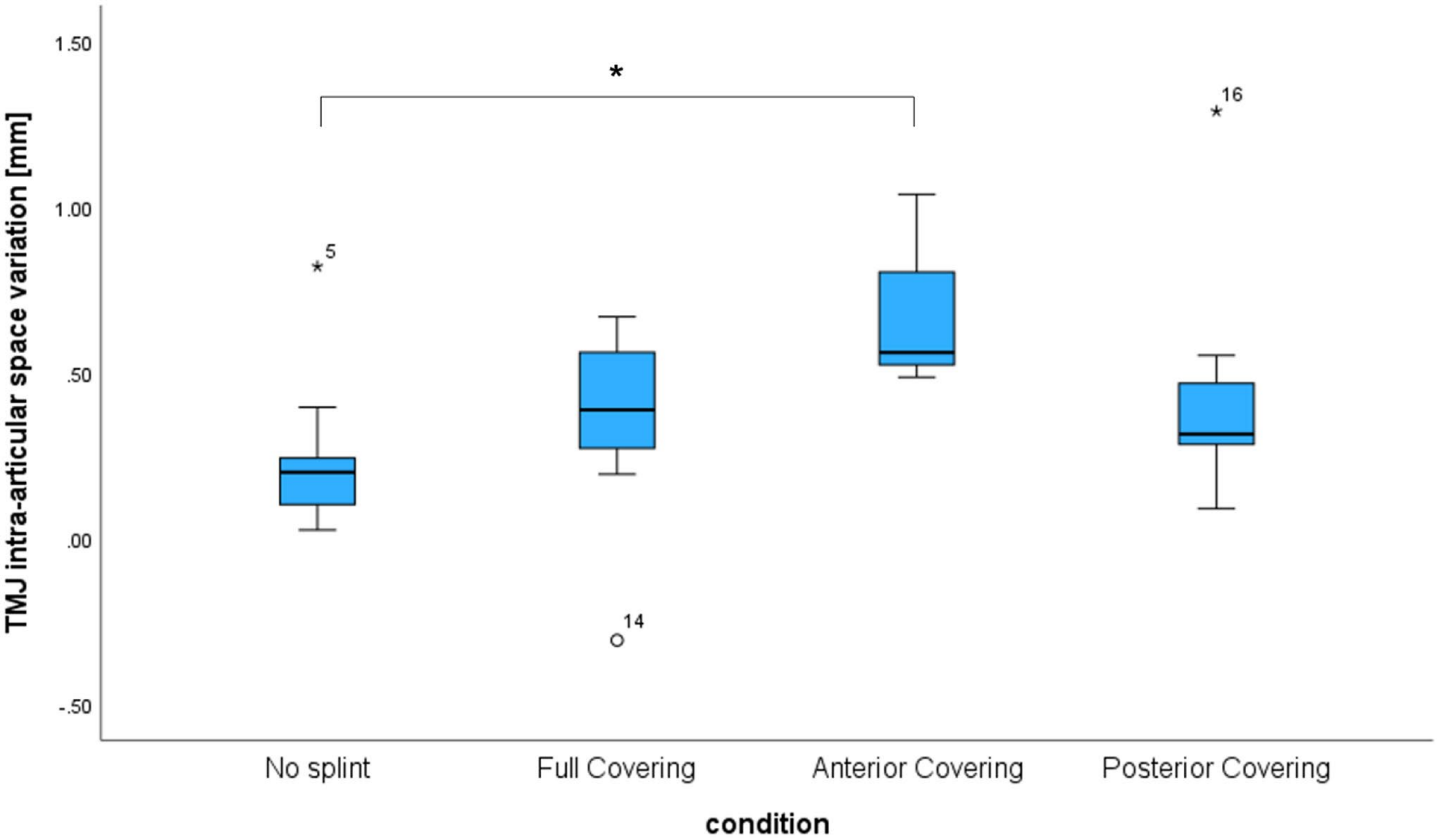
Graphic representation of the parameters describing the joint intra-articular space variation (ΔMID) of the joint between ICP and clenching with bite force of 100 N while wearing no splint, a full covering splint, an anterior bite splint, or a posterior bite splint

By splint insertion, an overall increase of MID was observed for full covering and posterior covering (*p* = 0.027 and *p* = 0.034, respectively). However, the clenching with 100 N on every splint reduced MID comparably to the condition without splint (Fig. 4). Clenching while wearing an anterior bite splint led to a ΔMID with respect to ICP almost three times larger than the no splint condition (*p* = 0.011) (Fig. 5, Table 1c). The clenching on no splint, the full covering splint and the posterior bite splint resulted in comparable ΔMID (*p* > 0.05).

## Discussion

The aim of this study was to analyze the variation of intra-articular space and EMG activity during ICP and clenching with 100 N while wearing three different occlusal splints (full covering splint, anterior bite splint, and posterior bite splint). To our best knowledge, this is the first study to compare the anterior bite splint to the full covering splint and posterior bite splint regarding change of MID whilst clenching. The results revealed that the anterior bite splint led to decreased EMG activation of the anterior temporalis muscle. This might be caused, on the one hand by the greater number and more sensitive periodontal mechanoreceptors in the incisors, which can lead to a lower maximal bite force. On the other hand, clenching on the anterior bite splint may lead to a changed mandibular position and therefore could lead to a stretching of the temporalis muscle along its main fibers [34–37]. This might have diminished the load and caused the reduction of the EMG. In contrary, the EMG of the masseter muscle showed similar changes in activation from ICP to clenching for all three splints and no splint. Previous studies stated that an anterior bite splint led to smaller EMG activation of the masseter muscle, which we could not reproduce in our study [38, 39].

All four conditions led to a reduction in MID during clenching, but only clenching on the anterior bite splint compared to no splint showed a statistically significant reduction. According to other studies, the insertion of occlusal splints led to an antero-caudal movement of the condylar reference point [28, 40]. The subsequent clenching on the splint resulted in an enhanced anterior and reduced caudal movement [40]. Therefore it can be inferred, that a repositioning of the MID occurred, which varied due to person-specific TMJ morphologies [28]. Since these findings only were made on occlusal splints covering the full dental arch and not on anterior bite splints, we only can assume that a similar movement of the mandible occurs wearing the anterior bite splint, considering, that we could not find significant differences regarding ΔMID in our study between the splints. Of particular concern is the large difference in the reduction of MID with the anterior bite splint, which was almost three times larger than the reduction without wearing a splint. Since the molars are not covered with the anterior bite splint, the clenching happened only on the front teeth, which could have resulted in a greater cantilever effect due to the anatomical position of the masseter muscle. This, together with the redistribution of MID in the joint, might be a reason for the big reduction of MID of the anterior bite splint while clenching. As there is a lack of studies on anterior bite splints and their mechanism of action, future investigations need to be done regarding their influence on the condyle-fossa relationship. Although large reduction in MID with the anterior bite splint was shown, it is important to mention, that the participants had great difficulties to even clench on their front teeth with 100 N and could only perform it for a few seconds. Therefore, we do not expect comparable results in the daily usage of the anterior splint, but even smaller EMG activities and smaller reduction of MID. The clenching without any splint led to a small reduction of MID, which might be due to the restricted space for the condyles to move cranially. One can assume, that the joint space is mainly influenced by the interarticular tissue, namely the articular disc and its poroviscoelastic properties [41]. It is therefore evident that the MID and its alteration during clenching varies inter- and intra-individually. The full covering and posterior bite splints appeared to have similar effects on the EMG and MID. This finding is in accordance to the study of Anton Demling et al. where no significant differences were found during clenching on two different occlusal splints (pivot and stabilization splint) by observing the movement of a condylar reference point [40]. It must be considered that the posterior bite selected for this study, the Aqualizer^®^, is filled with liquid and therefore soft and comfortable to bite on. Due to distribution of bite force this can easily lead to a higher bite force. Still, it should only be used as a temporary therapeutical device, because the liquid can be easily pressed out by material damage after high force clenching. In this study the splint was new and thus had a comparable thickness to the full covering splint. Therefore, the short-term use of the two splints did not reveal significant differences.

As the splint insertion leads to an increased vertical interocclusal distance [42], it can be assumed that an increased thickness of occlusal splints results in a more anterior and caudal position of the condyle [40]. To prevent a possible influence of the vertical dimension, both the full covering and anterior bite splint have been designed with the same thickness in the frontal region (1.5 mm). The prefabricated Aqualizer^®^ splint is available in three different thicknesses: 1 mm, 2 mm, and 3 mm. We used in our study the thickness of 2 mm in the premolar/molar region, and therefore a direct comparison to the other two thicknesses is not possible. Previous studies have shown that small variations in thickness (up to 1 mm) did not have an impact on the EMG of the masticatory muscles [43]. Considering this, we can assume that the thickness of the Aqualizer^®^ splint had no greater impact on the EMG. Since there was no significant difference in ΔMID wearing the three different splints, it can be assumed, that the thicker Aqualizer^®^ splint did not lead to a relevant change in the vertical interocclusal distance compared to the other two splints.

One major limitation of this study is the small sample size, due to the complexity of the data acquisition method. However, the results showed significant changes in the MID and EMG value of the temporalis muscles, which indicate that a sufficient power was reached for these two variables. Furthermore, one must consider that the data for this study was gathered from a group of asymptomatic and young subjects. The experimental analysis was conducted as cross-sectional study, not in longitudinal way, without considering further long-term effects of the device usage on the anatomical structures of the masticatory system. This might have masked possible adaptation effects on the musculo-skeletal level, that might arise with splint continuous use. However, the main goal of the study was an evaluation of the immediate effects and not a therapy response. An additional improvement of the study would require the enrollment of healthy and symptomatic volunteers and the evaluation of the outcomes at different time points.

Another important aspect to consider when interpreting the results is that due to the experimental setting and different splint design, despite the maximum attention posed to the preparation of the splints, the vertical dimensions of the three preparations might have been slightly different. Furthermore, as posterior-covering splint, the Aqualizer^®^ was selected, because it is standard and easily applicable. The mechanical properties of this splint are however different compared to a customized posterior splint as a pivot-splint or a distraction splint, that are usually made of hard resin and therefore more resistant. This choice might have influenced the bite force (since the resistance is lower) and the vertical dimension, that depends on the occlusal contacts.

Furthermore, technical limitations of the acquisition method, based on the digital 3D modeling of the TMJ, might play a role. The method does consider the mandible as a rigid body, without measuring the possible mandibular deformation due to bite forces and tooth intrusion during clenching and hence might be misinterpreted as condylar translation. Although in the study of Ting Jiang and Minoru Al it has been reported no significant deformation of the mandible (premolar area) during bilateral clenching in the canine area with 150 N [44]. Future studies will focus on splint insertion and shift in the position of the condyle in relation to the fossa on the one hand and to changes in the intra-articular load zone on the other, when habitual clenching and lateral grinding movements with force are performed.

## Conclusion

This study showed, within its experimental limitations, that differently constructed bite splints can have a different impact on the EMG of the masticatory muscles, and in MID. Especially the anterior bite splints seem to have a greater impact on the reduction of the muscle activation and therefore differ regarding their biomechanical effects from splints which cover the whole dental arch. We could demonstrate that the clenching on the anterior bite splints led to bigger reduction of MID and thus had the greatest influence on alteration in the condylar position.

## Data Availability

No datasets were generated or analysed during the current study.
